# SPAchips: Microparticles Used for the Selective In Vitro Labelling of Microglia

**DOI:** 10.3390/ijms26199773

**Published:** 2025-10-08

**Authors:** Justyna Gargas, Justyna Janowska, Beata Dabrowska-Bouta, Marta Sidoryk-Wegrzynowicz, Alberto M. Hernández-Pinto, Rubén Miguez, Teresa Suárez, Lidia Struzynska, Joanna Sypecka

**Affiliations:** 1 NeuroRepair Department, Mossakowski Medical Research Institute Polish Academy of Sciences, 5 Pawinskiego Str, 02-106 Warsaw, Poland; jgargas@imdik.pan.pl (J.G.); jjanowska@imdik.pan.pl (J.J.); 2 Laboratory of Pathoneurochemistry, Department of Neurochemistry, Mossakowski Medical Research Institute, Polish Academy of Sciences, 5 Pawinskiego Str, 02-106 Warsaw, Poland; 3 4cell Nanodevices SL. CAIT-UPM Campus de Montegancedo s/n, Pozuelo de Alarcón, 28223 Madrid, Spain; 4 Centro de Investigaciones Biológicas Margarita Salas (CIBMS-CSIC), C/Ramiro de Maeztu 9, 28040 Madrid, Spain

**Keywords:** astrocytes, microglia, neurons, oligodendrocytes, rat primary cultures, SPAchips^®^

## Abstract

Both basic and preclinical research, as well as the development of new therapies, require tools that allow for the selective labelling of specific cell types and the targeted delivery of drugs. The developed tools must then be validated in biological systems. In view of the lack of effective therapies for many neurodevelopmental disorders, including neonatal brain injuries, we decided to use the newly described, innovative SPAchips^®^ (a4cell, Pozuelo de Alarcón, Spain) tool and test it in labelling neonatal rat neural cells. In our studies, rat primary cultures of neurons and glial cells (astrocytes, oligodendrocytes, and microglia) were incubated with different concentrations of SPAchips^®^. At selected time points, uptake of the tested microchips by particular cell types was assessed using lineage-specific antibodies and visualized using a confocal microscope. Additionally, the potential cytotoxicity of added microparticles was verified, as was the possibility of microglia activation. The study indicates that the tested microdevices selectively label neonatal rat microglia and can be a useful tool for visualizing this cell type, as well as a non-toxic tool for developing innovative strategies based on the functionalization of microparticles aimed at modulating neuroinflammatory processes.

## 1. Introduction

In recent years, there has been intense development in the field of micro- and nanotechnology, which deals with the research of materials at the nanoscale. The physical and molecular properties of these materials differ significantly from those observed at a larger scale [1]. Advanced novel nano-based materials and innovative nano- and micro-scaled tools are being developed and are finding application in various areas of life, including human health and medicine [2,3,4]. A new interdisciplinary branch of nanotechnology has emerged, which is nanomedicine, combining biology, chemistry, engineering, and medicine. Its purpose is to provide new solutions in the diagnosis and treatment of cancer, neurodegenerative and cardiovascular diseases, and other problems and challenges of modern medicine [5]. The field of nano-based systems and nano/micro-scaled therapeutic tools such as imaging and diagnostic platforms, drug delivery systems, and materials for implants and tissue regeneration, is rapidly expanding [6].

A huge challenge for current medicine is, among others, the therapy of neurological disorders. The complex anatomy and physiology of the central nervous system (CNS) along with its unique anatomical and functional barrier, the blood–brain barrier (BBB), which separates brain tissue from systemic circulation, guarding a stable microenvironment in the brain parenchyma, as well as the limited regenerative capability of nervous tissue, are all major obstacles to treatment. The presence of the BBB complicates the diagnosis and treatment of CNS disorders due to restricting the tissue access of drugs and diagnostic and therapeutic substances [7].

In recent years, extensive efforts have been made in nanoscience to overcome these difficulties and achieve technological breakthroughs in the treatment of neurological disorders. A variety of nano- and microdevices exhibiting therapeutic or neuro-regenerative potential has been developed for neuro-diagnosis and neuro-therapy [8]. These tools are small enough to label, track, and operate in single living cells in an extra- and intracellular way. For instance, vertically aligned carbon nanofiber (VACNF)-based neural chips were designed to stimulate and record electrophysiological signals from brain tissue slices in vitro [9]. Furthermore, nanostructured opto-electronic devices have been found to effectively stimulate the light-insensitive chick retina, making them very promising in the development of artificial retinas and in restoring or improving vision in patients with ophthalmic disorders [10].

Certainly, there is still a need to conduct research on new solutions enabling the use of nanotechnology achievements in basic neuroscience and then in clinical neurology, as great expectations are placed on their profound impact on improving the diagnosis and treatment of neurological disorders. An example of novel nanostructured micro-tools are the suspended planar array chips (SPAchips^®^) previously introduced and described as useful for intracellular analysis of multiple biological parameters [11]. One of their most relevant features is the capability of being suspended, meaning that they can be released from the wafer and directly interact at the cell scale. These microdevices have been internalized by cultured HeLa cells where their potential to detect externally induced pH changes inside the living cells has been demonstrated; the possibility to use this tool for the multiplexed detection of intracellular biological parameters has been suggested.

This tool, found effective in HeLa cells, also seems to be an interesting solution for probing and manipulating other types of cells, including those of neural origin. Therefore, we are convinced that the specificity and sensitivity of SPAchips^®^ towards neural cells warrant thorough examination. Here, we focus on the biological application of this microdevice in terms of its functionality in neural cell research.

We performed studies using this technology in primary cultures of various neural cells of glial origin, such as microglia, astroglia, and oligodendroglia, as well as in neurons checking the ability of the chips to internalize. Additionally, the potential for internalization was demonstrated in a more complex 3D tissue system of retinal explants.

The results of our study indicate that SPAchips^®^ may be regarded as an attractive concept for studying molecular processes in living astrocytes and microglia at the level of a single cell.

## 2. Results

**Figure 1:**
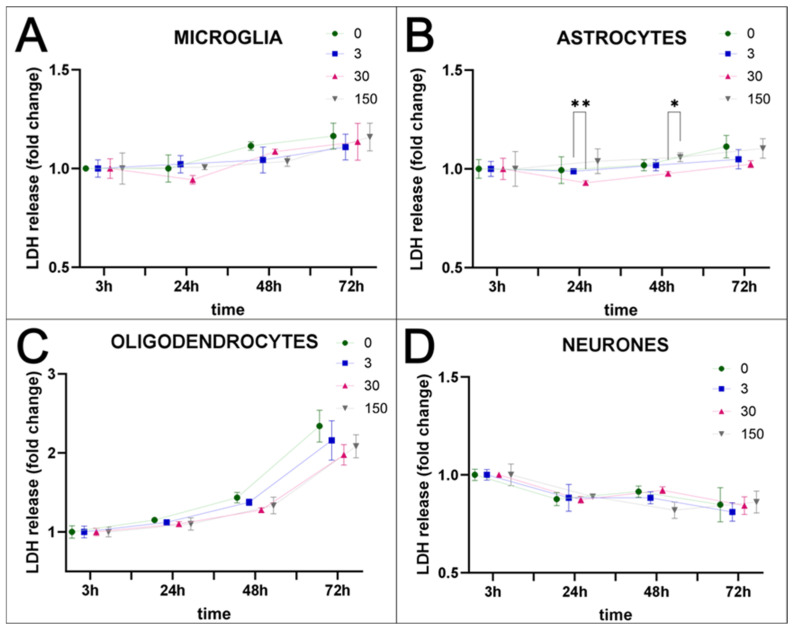
Cytotoxicity assay–time- and concentration-dependent release of LDH. Incubation of glial and neuronal cells with SPAchips^®^ in different concentrations (0–150 × 10^3^ per well) did not have a negative effect on cell viability. Whenever SPAchips^®^ spontaneously enter or are phagocytosed by microglia (**A**), or do not directly interact with astrocytes (**B**), oligodendrocytes (**C**), or neurons (**D**), they are never cytotoxic. The values of absorbance reflecting the released LDH level normalized to the measurements at 3 h after starting incubation with SPAchips^®^ for each concentration are never significantly different from control cultures. For statistical analysis, normal distribution was tested with the Shapiro–Wilk test. The differences in the obtained results were evaluated by two-way ANOVA. The only factor significantly affecting the results was time of incubation (DFn = 3, DFd = 24). Time accounted for 59.4% of the total variance in microglia (*p* < 0.0001; F = 24.53); 30.99% in astrocytes (*p* = 0.0033; F = 9.68); 93.81% in oligodendrocytes (*p* < 0.0001; F = 512.45); and 67.22% in neurons (*p* < 0.0001; F = 40.43). Several statistically significant differences were observed for multiple comparisons for different SPAchips^®^ concentrations in astrocytes and are shown in the graphs (* *p* < 0.05; ** *p* < 0.01).

Nanoparticles could serve as a very useful tool to stain the selected cells within body tissues, especially those fluorescently labelled, which facilitate the identification of cells within examined tissues. To check the utility of SPAchips^®^ in studying cells of the nervous tissue, naïve, non-passaged and non-stimulated primary neural cells were incubated in vitro with two different concentrations of these chips (Figure S1A). To recreate in vitro physiologically normoxic conditions of the brain nervous tissue, cells were cultured in strictly controlled environmental conditions, corresponding to 5% O_2_ and 5% CO_2_. To avoid stimulation or potential protection by undefined factors, cells were kept in a serum-free media during the entire period of cell co-culturing with the tested nanoparticles. The very first observations on the interaction of the chips with cells were performed on cultures labelled with Hoechst and observed in fluorescence and transmitted light (Figure S1B–E). Numerous red-labelled SPAchips^®^ of characteristic shape were found in the ramified, non-stimulated microglial cells (Figure S1B,C). Contrary to the chips found in microglial cells, single chips were observed very close to astrocyte cells (Figure S1D), and some were also attached to cultured neurons (Figure S1E); however, their exact localization on the cell membrane should be further confirmed with selective membrane tracers. Cultured cells did not change their morphology during incubation with SPAchips^®^, nor did they induce condensation of chromatin and nuclear shrinkage. This suggests that SPAchips^®^ are not cytotoxic to the neural cells. This observation was further confirmed by biochemical assay based on the measurement of the activity of the lactate dehydrogenase (LDH), which is released from the cytosol of damaged cells into the culture medium. The tested concentrations of nanoparticles corresponded to either 3, 30, or even as much as 150 × 10^3^ SPAchips^®^ per well. LDH activity was measured in supernatants of all of the examined cultures of neural cell fractions at 3, 24, 48, and 72 h of incubation (Figure 1A–D). According to two-way ANOVA of the obtained data, the only factor significantly affecting the results was the time of incubation. Statistically significant differences were observed for multiple comparisons for different SPAchips^®^ concentrations in astrocytic fraction only at 24 h (** *p* < 0.01) and at 48 h (* *p* < 0.05; Figure 1B). However, at the later time point, i.e., at 72 h of incubation, the potential differences were not statistically significant; thus, they do not represent a significant trend.

### 2.1. SPAchips^®^ in Microglia Culture

**Figure 2:**
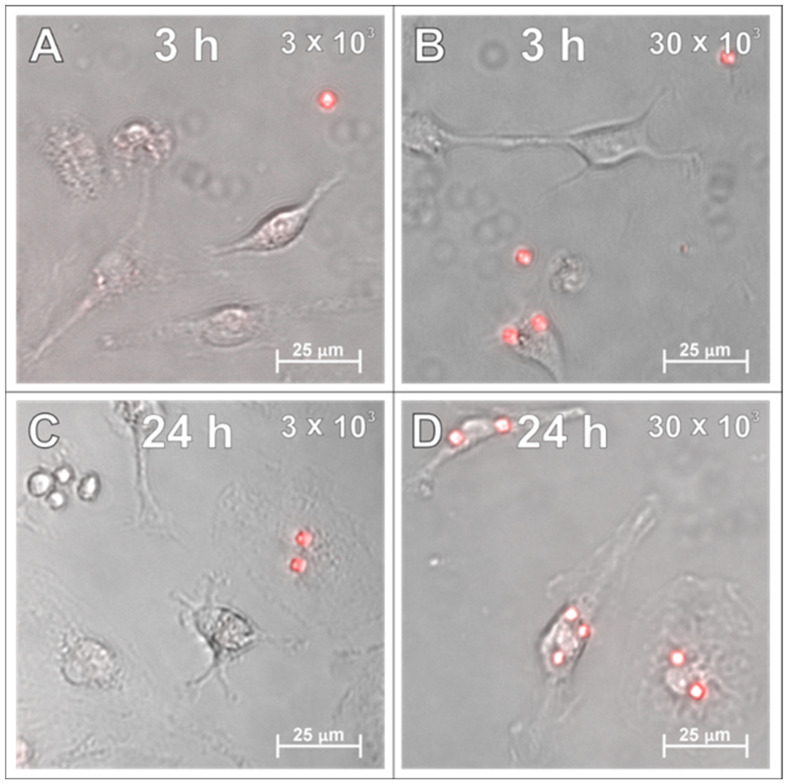
Live observations of microglia incubated with either 3 × 10^3^ (**A**,**C**) or 30 × 10^3^ (**B**,**D**) SPAchips^®^ per 8 × 10^4^ cells, after 3 h (**A**,**B**) and 24 h of incubation (**C**,**D**). Cultures observed in Cell Observer system (Zeiss) under magnification 40×. Scale length in image corresponds to 25 µm.

**Figure 3:**
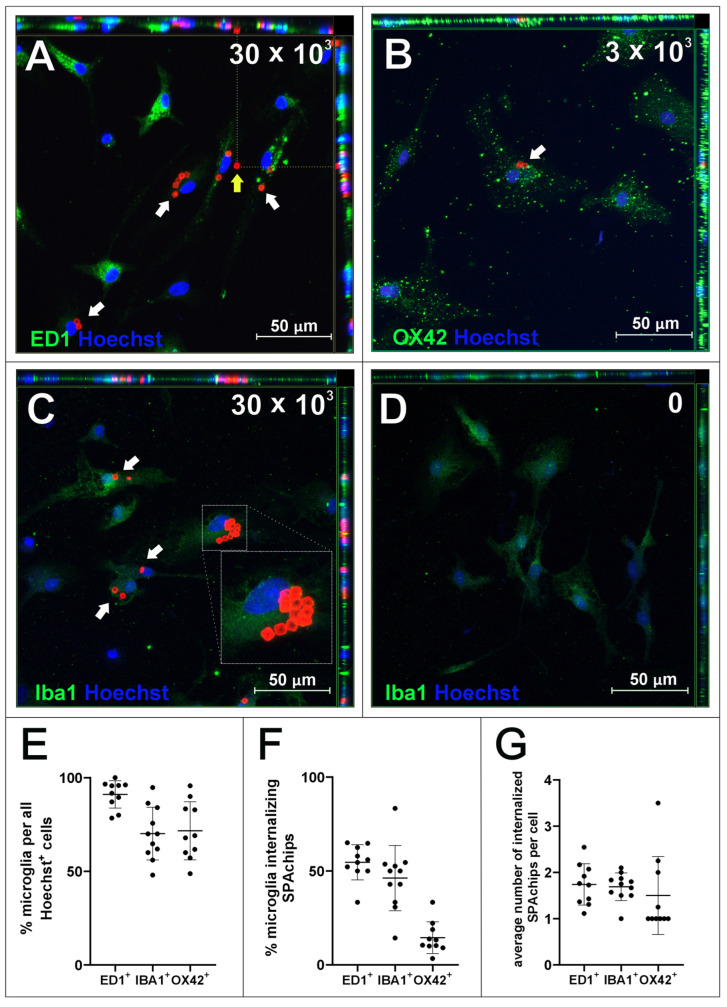
Microglia cell cultures incubated with either 30 × 10^3^ (**A**,**C**) or 3 × 10^3^ (**B**) microchips per 4 × 10^4^ cells, control cultures without chips (**D**), fixed after 48 h of incubation. Cultures were labelled with Hoechst to visualize cell nuclei as well as with antibodies for typical markers of microglia/macrophages: ED1 (**A**), OX42 (**B**) and Iba1 (**C**,**D**). White arrows indicate chips inside cells. Yellow arrows indicate chip outside cell. Z-stack images were collected with Zeiss LSM780 confocal microscope under magnification 40×. Scale length in image corresponds to 50 µm. (**E**–**G**) Counting of ED1+, Iba1+, and OX42+ cells in isolated microglial cultures. (**E**) Positively labelled cells constituted percentage of all Hoechst+ cells in the culture. (**F**) Percentage of microglia cells identified by selected markers that internalized at least 1 SPAchip. Concentration of SPAchips added to microglia cultures was 30 × 10^3^ SPAchips per 4 × 10^4^ cells for ED1 and Iba1 labelling or 3 × 10^3^ SPAchips per 4 × 10^4^ cells for OX42 labelling. (**G**) Average number of SPA chips internalized by microglial cells identified by selected markers. (**E**–**G**) Cell counting was performed on randomly selected 10 fields of view (black dots).

In the next set of experiments, the SPAchips^®^ were added to the microglial cultures in two different concentrations to check if the uptake occurs in a dose-dependent manner. Live recording of cells after 3 and 24 h of incubation with nanoparticles in both the low (Figure 2A,C) and high (Figure 2B,D) concentrations allowed us to observe the co-localization of SPAchips^®^ within the cell body of microglia. To precisely describe the localization of nanoparticles, microglia were stained with lineage-specific antibodies (OX42, ED1, IBA1) and subjected to analyses by means of confocal microscopy. Acquisition of the images in the Z-stack option allowed us to confirm the intracellular localization of SPAchips^®^, which are always close to the cell nuclei (indicated with white arrows, Figure 3A–C). One cell could phagocyte up to a dozen of chips (white square, Figure 3C). The yellow arrows indicate that the SPAchip was found attached to the cell membrane outside the cell (Figure 3A). Iba1 labelling performed in cultures after incubation with high concentrations of chips (Figure 3C) and in control cultures (Figure 3D), without chips, confirm that these particles did not induce the activation of microglia (no change in Iba1 fluorescence intensity), nor did they induce changes in cell morphology.

Counting cells labelled with different microglial cell markers showed a higher percentage of ED1+ cells than cells labelled with Iba1 or OX42 in the culture (Figure 3E). The proportion of cells internalizing SPAchips was higher at high concentrations (in ED1+ and Iba1+ cells) than at low concentrations of SPAchips added to the culture (in OX42+ cells; Figure 3F). However, the average number of internalized chips per cell was similar. In microglia cultures, no internalization of SPAchips by cells not labelled with any of the markers used was observed.

### 2.2. SPAchips^®^ in Oligodendrocyte Culture

**Figure 4:**
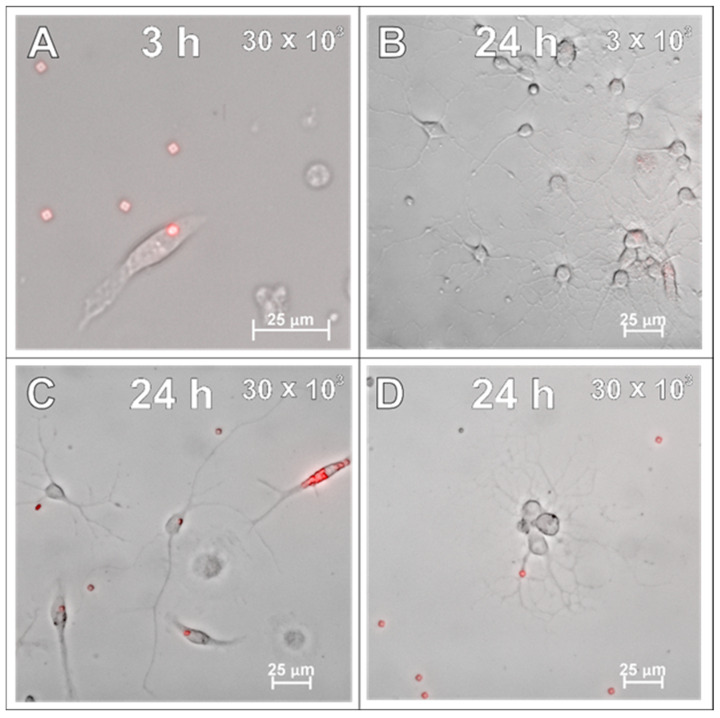
Live observations of oligodendrocytes incubated with either 30 × 10^3^ (**A**,**C**,**D**) or 3 × 10^3^ (**B**) SPAchips^®^ per 8 × 10^4^ cells, after 3 h (**A**) and 24 h of incubation (**B**–**D**). Cultures observed in CellObserver system (Zeiss) under magnification 40×. Scale length in image corresponds to 25 µm.

**Figure 5:**
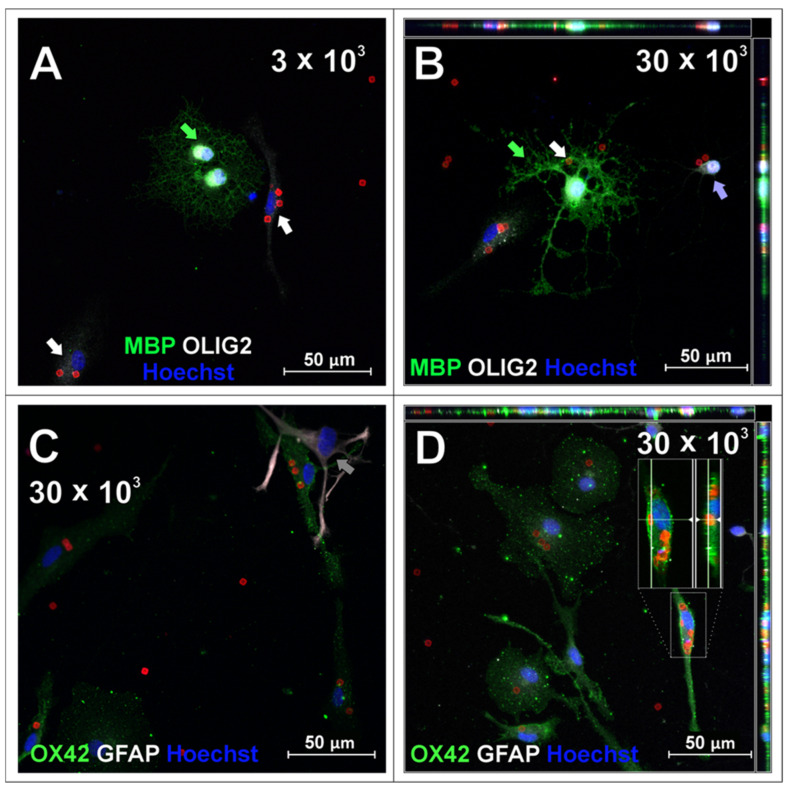
Oligodendrocyte cell cultures incubated with 3 × 10^3^ (**A**) or 30 × 10^3^ (**B**–**D**) chips per 4 × 10^4^ cells, fixed after 48 h of incubation. To visualize oligodendrocyte lineage cells, cells were labelled with anti-MBP and anti-OLIG2 antibodies. Green arrows indicate OLIG2+ (nuclear)/MBP+ cells identified as mature oligodendrocytes, purple arrows indicate cell identified as oligodendrocyte progenitor OLIG2^+^ (nuclear)/MBP^−^. Cells that were present in oligodendrocyte cell cultures that had different morphologies and are common impurities after isolation with described method, confirmed with immunofluorescent labelling, were identified as microglia (OX42-positive, green, **C**,**D**) and astrocytes (GFAP-positive, white, **C**). White arrows indicate cells identified as microglia, grey arrows indicate cell identified as astrocyte. Purple arrow points to oligodendrocyte progenitor cell. Z-stack images were collected with Zeiss LSM780 confocal microscope under magnification 40×. Scale length in image corresponds to 50 µm.

In parallel with the microglial culture, the potency of SPAchips^®^ to label the brain macroglia was checked as well. Accordingly, SPAchips^®^ were added in both the low and higher concentrations to the cultures of OPCs, which were differentiating towards the mature oligodendrocytes, capable of carrying out the myelination process. During their differentiation, the small, bipolar cells elaborate long, highly ramified cell processes. The complexity of their morphology makes oligodendrocytes interesting cells to study, including at the nanoscale; this is based on their small progenitors, noting that immature oligodendrocytes only have few cellular extensions to the cells subjected to highly branched processes that search for axons to be myelinated. Live observations of OPCs cultured for 3 h with SPAchips^®^ indicated that there might be cells capable of internalizing SPAchips^®^ (Figure 4A). However, with the longer incubation time, differentiating oligodendrocytes identified with branched morphology did not have SPAchips^®^ within the cell body (Figure 4B), except for a few cells with a typical morphology (Figure 4C), and some were attached to the oligodendrocyte cell extensions (Figure 4C,D). Therefore, oligodendrocyte cultures were carefully examined by means of confocal microscopy in the context of the SPAchips^®^ presence (Figure 5A,B), as well as possible contamination of the oligodendrocyte cell culture with other residual glial cells after the isolation procedure (Figure 5C,D). To confirm the cell phenotype, the highly specific antibodies against the Myelin Basic Proteins (MBP typical for oligodendrocytes as the only cells within CNS capable of biosynthesizing and elaborating the myelin sheaths) and OLIG2 (the transcriptional factor characteristic for oligodendroglial lineage, exhibiting nuclear localization in oligodendrocyte progenitors) were used.

Oligodendrocyte marker OLIG2 was found within the cell nuclei of mature, MBP-positive oligodendrocytes (green arrows, Figure 5A,B) as well as, presumably, oligodendrocyte progenitors (Figure 5B, purple arrow), characterized by bipolar morphology in culture. SPAchips^®^ colocalized with the body of the cell of unknown phenotype (Figure 5A, white arrow); however, they are not present neither in the cytoplasm nor in the cell extensions of oligodendrocytes (Figure 5A,B). Both oligodendrocyte progenitors and mature oligodendrocytes did not internalize any chip; however, oligodendrocyte processes seemed to seamlessly wrap or cover a chip attached to the coverslip (Figure 5B, white arrow). Cells that were present in oligodendrocyte cell cultures that had different morphologies and are common contaminants were identified as microglia (OX42-positive, green, (Figure 5C,D) and astrocytes (GFAP-positive, white, Figure 5C). Apparently, microglia remained the only glial fraction that spontaneously phagocytosed the chips, clearing them from other cells in the culture.

### 2.3. SPAchips^®^ in Astrocytes Culture

**Figure 6:**
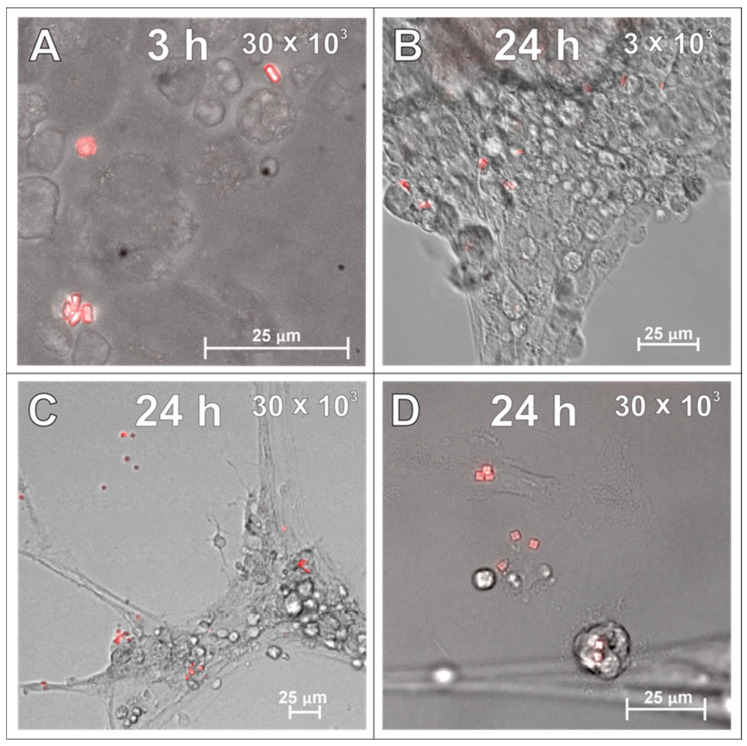
Live observations of astrocyte cultures supplemented with two different concentrations of SPAchips^®^ at various time points from initiation of incubation: either 3 (**A**) or 24 h (**B**–**D**), by means of CellObserver system (Zeiss) and under magnification 40×. Scale length in image corresponds to 25 µm.

**Figure 7:**
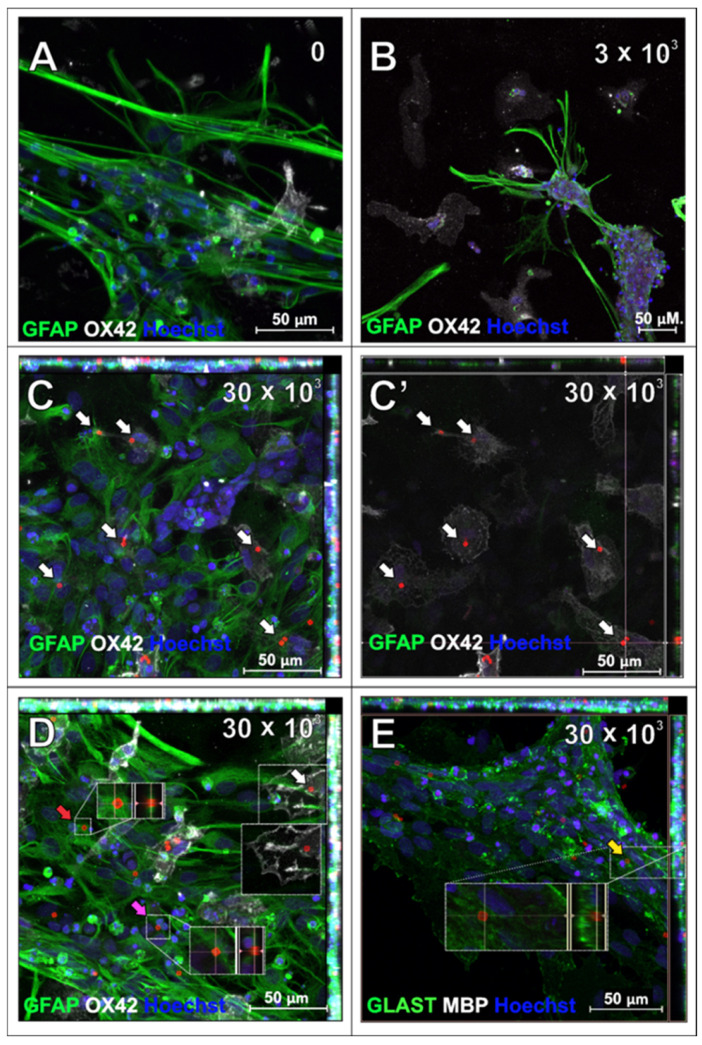
Astrocytes which have been incubated with SPAchips^®^ for 48 h to examine their internalization within cell body: untreated control culture (**A**), cultures supplemented with either 3 × 10^3^ (**B**) or 30 × 10^3^ (**C**–**E**) SPAchips^®^ particles. (**C**,**C’**) Single plane focused at level of OX42-positive microglia. Cells were labelled with astrocytic markers (GFAP-green; (**A**–**D**); or GLAST-green; （**E**)) and additionally with either microglial (OX42, white; (**A**–**D**)) or oligodendroglial (MBP, white, not found in cultures; (**E**)) cell markers. Hoechst was used to visualize cell nuclei. White arrows indicate SPAchips^®^ inside cells identified as microglia with OX42 labelling. Yellow arrow: Microchips visible under cell extensions. Red arrow: SPAchips^®^ found close to astrocytes, presumably were attached to cell surface. Pink arrow: Microchips localized between astrocytic cells. Images were collected with application of Zeiss LSM780 confocal microscope under magnification 40×. Scale length in image corresponds to 50 µm.

The primary rat astrocytes were the second macroglial fraction studied in the context of SPAchips^®^ uptake. The first observations of the live astrocyte cultures supplemented with two different concentrations of nanoparticles indicated their presence in the cytoplasm of the cultured cells, both at 3 h after co-incubation (Figure 6A) and at 24 h after supplementing the culture media with SPAchips^®^ (Figure 6B–D). Astrocyte phenotype was confirmed by immunostaining the cells with specific antibodies directed against either Glial Fibrillary Acidic Proteins (GFAP; Figure 7A–D; green) or against the astrocyte cell surface antigen-1 (ACSA-1/GLAST; Figure 7E).

Incubation with SPAchips^®^ did not affect astrocyte viability, morphology, or GFAP immunofluorescence quality compared to the control culture (Figure 7A). After the fixing and immunolabelling procedure, most of the chips added in a low concentration were washed out, suggesting that they did not enter these cells (Figure 7B). In high concentrations, SPAchips^®^ were found in fixed cultures, which we can observe in Z-stack maximum intensity projection images; however, immunostaining with OX42 marker confirms that most of the internalized SPAchips^®^ are present within the cytoplasm of microglia (Figure 7C,D). This was confirmed on the single plane focused at the level of OX42-positive microglia (white arrows in Figure 7C and Figure 7C’, respectively). Only a few SPAchips^®^ were found close to astrocytes but presumably were attached to the cell surface (Figure 7D, red arrow), localized between the cells (Figure 7D, pink arrow), or under the cell extensions (Figure 7E, yellow arrow). To confirm the astrocyte phenotype, an astrocyte-specific anti-glutamate/aspartate transporter antibody, GLAST, was used (Figure 7E, green). No MBP-positive oligodendrocytes were found in these cultures (Figure 7E).

### 2.4. SPAchips^®^ in Neuron Culture

**Figure 8:**
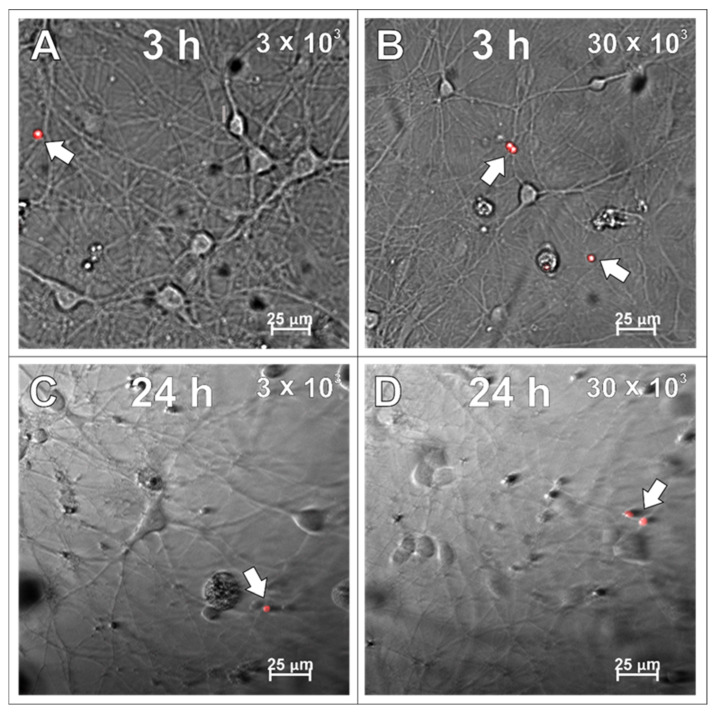
Live observations of neurons incubated with 3 × 10^3^ (**A**,**C**) or 30 × 10^3^ (**B**,**D**) chips per 8 × 10^4^ cells, for 3 h (**A**,**B**) and 24 h (**C**,**D**), respectively. White arrows indicate chips on the surface of the neuronal culture. Cultures observed in CellObserver system (Zeiss) under magnification 40×. Scale length in image corresponds to 25 µm.

After examining the glial cells of the developing brain, which are all supportive of neurons and guarantee the proper, physiological functioning of the nervous tissue, the primary cultures of neurons were studied in the context to be labelled with the applied SPAchips^®^. Similarly to what was verified on glial cells, live observations were also performed on neurons cultured with SPAchips^®^ administered in two doses. Thanks to the live recording of the cells with the use of the confocal CellObserver system at 3 (Figure 8A,B) and 24 h (Figure 8C,D), after applying SPAchips^®^ in the two tested doses, their localization on the surface of the neuronal cultures was noted. This system also allowed us to visualize cultured cells within the first hours after supplementing culture media with the tested factors and to confirm that the experimental variant is morphologically comparable to the untreated controls. 

**Figure 9:**
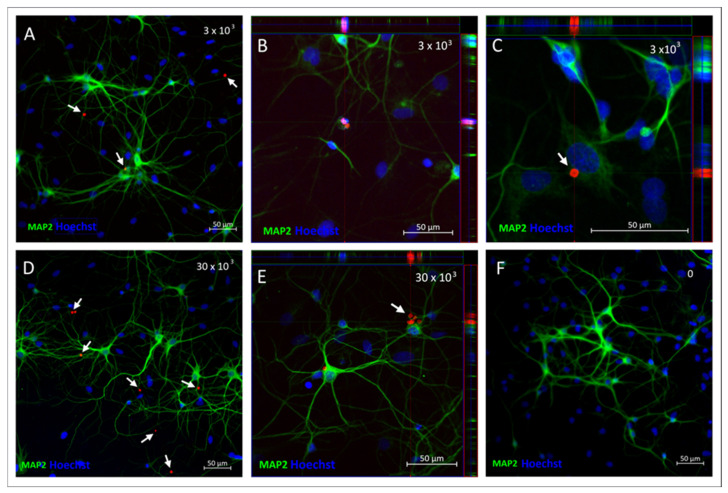
Neuronal cultures incubated either with 3 × 10^3^ (**A**–**C**) or 30 × 10^3^ (**D**,**E**) SPAchips^®^, control culture without chip addition (**F**), fixed after 48 h of incubation. MAP-2 antibody (green) was used to stain mature neurons. White arrows indicate presence of SPAchips^®^ in primary neuronal culture Hoechst dye was used to stain cell nuclei (blue). Z-stack images were collected with Zeiss LSM780 confocal microscope under magnification 40×. Scale length in image corresponds to 50 µm.

The above observations, as well as the neuronal phenotype of the cultured cells, were confirmed by their immunostaining with the antibody recognizing Microtubule-Associated Protein 2 (MAP2) 48 h after incubation with either 3 × 10^3^ (Figure 9A–C) or 30 × 10^3^ (Figure 9D,E) of SPAchips^®^ and compared to the control neuronal culture (Figure 9F). The Z-stack recording allowed us to check the SPAchips^®^ location in the very close vicinity to the cells; however, examination of several microscopic slides indicate that they are located extracellularly.

### 2.5. SPAchips^®^ as Tool to Stain Cells of Retina Explants

**Figure 10:**
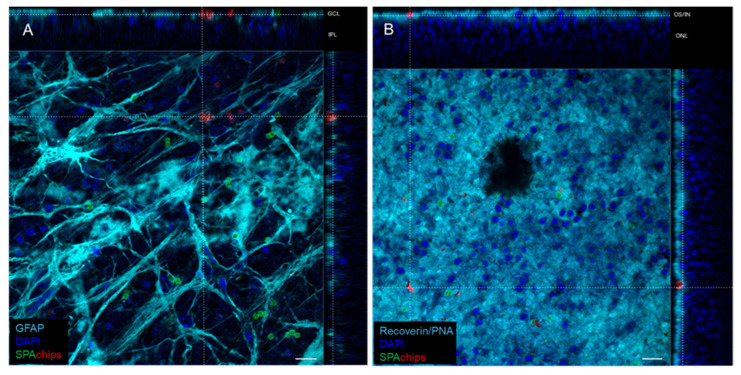
Representative orthogonal projections from confocal images showing SPAchip^®^ cellular uptake in whole-mounted retinas from mice. Chips functionalized with AlexaFluor^TM^-488 or CF^TM^-568 fluorophores were incubated for 72 h on retinal explants photoreceptor side-down (**A**) or -up (**B**). (**A**) On Ganglion Cell Layer (GCL), chips were mainly internalized by astrocytes (labelled with anti-GFAP antibody) yet barely reached inner plexiform layer (IPL). (**B**) On outer retina, chips could not pass through external limiting membrane, but accumulated on outer/inner segment (ON/IS) layer, and it was not possible to elucidate whether they were internalized or not by photoreceptors. Scale bar = 15 µm.

To check the SPAchips^®^ distribution and cellular uptake within a differentiated nervous tissue, we used retinal explants as a model. Retinas were whole-mounted photoreceptor-side up or down and SPAchips^®^ were added and incubated 72 h before fixation. Surprisingly, immunofluorescence images showed that microparticles were mainly internalized by astrocytes in the Retinal Ganglion Cell layer (RGC) (Figure 10A). On the other hand, SPAchips^®^ were easily found in the outer/inner segment (OS/IS) layer, but they could not pass through the Outer Limiting Membrane of the photoreceptors.

Besides that, it was not possible to demonstrate microparticle internalization by photoreceptors. Thus, while in the RGC, most of the chips were associated with GFAP (astrocyte marker), in the OS, chips seem to be imbricated in the tissue, stained by recoverin antibody and PNA, yet are not clearly internalized in rods or cones (Figure 10B).

## 3. Discussion

The nervous tissue of the CNS is composed of several cell types enabling its efficient functioning through the entire lifespan [12,13]. Although neurons are the major cell type responsible for nervous signal transduction, their survival and functioning are extensively supported by the specialized glial cells. Axon insulation by the highly compacted, lipid-rich myelin sheaths, enabling fast salutatory signal transduction, is elaborated by the cell processes of oligodendroglia [14,15,16]. On the other hand, astrocytes perform multiple functions, such as the biochemical control of cells that form the blood–brain barrier, and trophic support to neurons and tissue repair or scarring following infection or damage; they also participate in neurotransmission as an element of the tripartite synapse [17,18,19].

While oligodendrocytes and astrocytes are the major supporters of neuronal functions and keepers of biochemical tissue homeostasis, microglia are recognized as attentive brain guardians. In their resting form, they survey the surrounding microenvironment [20,21]. When activated by pathophysiological clues, they retract their processes, become phagocytic, and change their secretory profile to communicate with the other cell types by elevating or downregulating the expression of cytokines (especially different classes of chemokines) and trophic factors or releasing the classical signalling molecules associated with the inflammatory process [22,23,24]. Accordingly, the factors secreted by so called the M1 (pro-inflammatory) phenotype including interleukins (IL-1β, IL-6, IL-17, IL-18, IL-23, TNF-α) and a plethora of chemokines (including CCL5, CCL20, CXCL1, CXCL9, and CXCL10 acting as chemoattractants), also signal molecules like inducible nitric oxide synthase (iNOS), reactive oxygen species (ROS), the complement component c1q, and prostaglandins [25]. They are potent to induce the cell response of both astrocytes and oligodendrocytes, amplifying changes that occur to the affected tissue [26,27]. Since microglia are therefore a major contributors to the prolonged neuroinflammatory process and resulting neurodegeneration, clinical strategies targeting this cells are developed, aiming at changing microglia polarization from the M1 to M2 (i.e., anti-inflammatory) phenotype or using microglia replacement therapy [28,29,30,31,32]. The latter is characterized by the expression of pro-regenerative factors like TGF-β, IL-10, IGF-1, glial-derived neurotrophic factor (GDNF), BDNF, and panel of other chemokines (e.g., CCL13, CCL14, CCL17, CCL18, and CCL22). The polarization of microglia restrains their contribution to degenerative processes and due to the changes in their secretory activity, they modulate the local tissue microenvironment and restore tissue homeostasis [33,34].

Although many of the functions performed by glial in the CNS are intrinsic to its development and physiological functioning, they are all recognized as supportive for neurons, which are responsible for the efficient and rapid transduction of nervous signals. Due to the high energy demands associated with simultaneous transduction of multiple signals, they uptake the energy substrates from the extracellular space, but also sense the clues released to the local microenvironment [35,36,37,38]. Lack of trophic support or even insulation of the axon of moderate or large calibre by myelin sheaths can trigger axon degeneration. Yet the necessity of securing neuronal functions makes neurons relatively resistant to the influence of clues or signals which are not conveyed via synaptic transmission. It is however not a perfect mechanism, as demonstrated by a spectrum of neurodegenerative disorders. However, in this context, the lack of active uptake of the relatively large microparticles by rat neurons can be associated with physiological resistance to protect their functioning.

The plethora of pathological conditions affecting the nervous system exerts detrimental effects on the cell functioning, including (even predominantly) neurons. The first line of defence engages microglia, as the cells are able to polarize between the M2 (silent) to M1 (pro-inflammatory) phenotypes. The latter, associated with so called ameboid morphology and phagocytic capacity, is able to eliminate the objects (viral or bacterial pathogens, cell debris, foreign bodies, protein aggregates) present in the extracellular space. To combat the pathophysiological conditions, microglia that were activated change their secretory activity towards release pro-inflammatory factors, which also serve as signalling molecules for other cell types, including oligodendrocytes, astrocytes, neurons, or cells directly associated with immunological response, resulting in neuroinflammation accompanying the majority of disorders occurring to the CNS [39]. As microglia are recognized as the cells governing the process, they are the target of potential clinical strategies aimed at restraining disease progress towards neurodegeneration. Therefore, the therapies aimed at modulating microglia polarization between pro-inflammatory and M2 phenotypes are thought to be beneficial for promoting neurorestorative processes. There is however the urgent need to develop cell-specific treatments to target microglia. The functionalization of micro- and nanoparticles selectively uptaken by the major players of neuroinflammatory processes can be a promising strategy. A wide range of nanoparticles, including silica, magnetic, and gold nanoparticles; liposomes; carbon nanotubes; quantum dots; upconverting nanoparticles, among many others, have been tested in diverse biological systems. However, as there are still no treatments for many brain disorders, new tools have yet to be developed [40,41,42,43].

The combination of planar array (PA) and suspended array (SA) technologies enabled the development of innovative suspended planar array (SPA) chips. The resulting microparticles offer the advantages of both SA and PA, enabling the preservation of advanced fluid-phase kinetics and the multiplexed detection of intracellular biological parameters via a single device. SPAchips technology also opens up multiple ways for surface functionalization, which is of significant value in strategies aimed at developing targeted drug delivery systems [44]. The SPAchips dimensions, which were fixed to 3 × 3 × 1 μm^3^, were calculated to occupy only about 0.35% of the total volume of human cells. Those parameters allowed for the assessment of the microchip’s internalization by neural rat cells. As many preclinical approaches are based on in vitro, ex vivo, and finally in vivo studies on animal models of selected diseases, the studies testing microdevices in animal-derived in vitro systems allows for checking their utility in further studies towards developing clinical strategies.

Accordingly, many of the nanoparticles that have been widely used in various laboratory approaches to date have been reported to have a neurotoxic effect. Silver nanoparticles are probably the most well-known in nanomedicine and cosmetics. However, they have been proven to negatively affect rat cerebral astrocytes and hippocampal cells [45,46,47], liver and endothelial cells [48,49], and cardiomyocytes [50] by disrupting cellular homeostasis due to oxidative stress, increasing caspase activities, causing mitochondrial dysfunctions, and leading to apoptosis. Similarly, zinc oxide nanoparticles, as well as other metal-containing nanocarriers, were shown to exert toxic effects on neural cells of the central nervous system, due to different mechanisms involving oxidative stress and resulting in DNA and mitochondrial damage, the modulation of cell secretome, and the initiation of neuroinflammation and often cell loss due to the occurrence of pathophysiological conditions [51,52,53].

In this context, dose-dependent studies have to be carried on to test the new innovative microdevice to be used in biological systems. The study performed on all of the four major types of neural cells of fragile, developing brains indicate that both the tested SPAchips^®^ doses (even as high as 1 per 30 cells, which is possible to be modelled in vitro) are not cytotoxic to the examined neural cells. The study pointed out that the microparticles are selectively uptaken by the microglia in CNS-derived primary cells and in retinal explants, where the results suggest an active uptake by the GCL astrocytes. In this regard, it has been suggested by other authors that, due to the special composition and physiology of the retina (lack of oligodendrocytes or significant oxygen consumption) in comparison with other parts of the CNS, retinal astrocytes might be forced to develop new unexpected functions when compared with white-matter astrocytes [54], which may explain differences in SPAchips^®^ uptake between retinal astrocytes and CNS-isolated astrocytes. Furthermore, when cells were in their natural tissue environment, astrocytes also showed limited phagocytic capacity under certain conditions [55,56,57,58].

Our studies on neonatal rat neural cells, which are especially vulnerable to the external clues present in the extracellular compartments in this particular neurodevelopmental period, show that the tested microparticles are safe and do not induce changes in cell morphology or membrane leakage. Among all the four tested types of neural cells, LDH release was relatively increased in oligodendroglial cultures. Cultured oligodendrocytes, which are seeded as the progenitor cells, quickly differentiate in vitro, especially under serum-free conditions. Therefore, in all the experimental variants, at the 72 h time point, the LDH increases, which corresponds to the elaboration of the multibranched oligodendroglial processes, while a part of oligodendrocytes undergo either cell lysis or senescence, both in control and SPAchips^®^-treated cultures.

The delivery of nanoparticles targeted at microglia has previously been reported by a few laboratories. Given the huge need for broad applications, new tools are constantly being developed [59,60,61,62]. The selective uptake of tested microparticles by microglia/macrophages is a very promising observation in the context of preclinical studies and developing targeted therapies in many neurodevelopmental disorders [44,63,64], as well as neurodegenerative diseases, accompanied by inflammatory processes [65,66]. Various types of nanoparticles have already been used in different approaches to combat neurological deficits associated with the progression of Alzheimer’s or Parkinson’s diseases [67,68,69]. Another therapeutic target of microglia-oriented strategies is brain tumours, including aggressive gliomas [68,70,71,72]. Considering that efficient therapies for the aforementioned brain diseases are still lacking, there is an urgent need to develop new tools that can be applied in innovative strategies.

To be concluded, the SPAchips^®^ can be a useful non-toxic tool for the development of innovative strategies based on microparticle functionalization aimed at the modulation of neuroinflammatory processes. While at the initial period of pathology occurring to CNS, microglia activation is beneficial for protecting tissue (like the elimination of microbial pathogens, clearing tissue from debris resulting from necrotic cells degradation, promoting other cell activation like, e.g., oligodendrocyte differentiation, etc.) the lasting, prolonged neuroinflammation can contribute to the progress of neurodegeneration. The therapies applied in the secondary phase of development of inflammation may restrict microglia over-reactivity and contribute to its polarization towards phenotype secreting factors which promote tissue restoration. Thus, further studies on the SPAchips^®^ functionalization are encouraged to develop innovatory strategies for the selected CNS disorders.

## 4. Materials and Methods

### 4.1. Manufacturing and Labelling of SPAchip^®^

**Figure 11:**
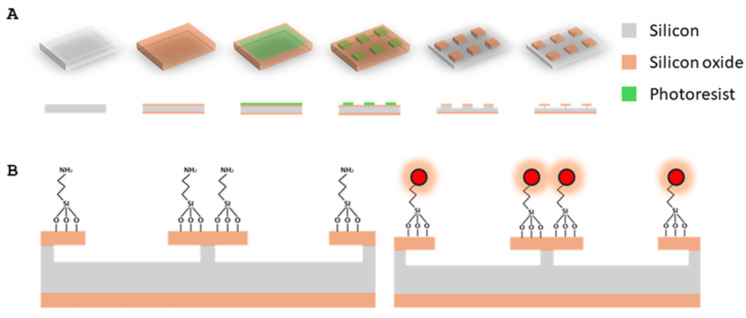
(**A**) Detailed scheme of fabrication of bare SPAchips: 1-μm silicon oxide layer is grown onto silicon wafer. Then, 3 × 3 μm lateral dimensions are defined by photolithography. Exposed silicon oxide is etched by dry-etching and, finally, after phororesist stripping, RIE step defines anchor beneath each chip. (**B**) SPAchips functionalization by silanization with APTES and fluorophore immobilization through amino-succinimidyl reaction. Modified from Torras et al. (2016). [11].

SPAchips^®^ wafers were manufactured by D+T Microelectrónica (Barcelona, Spain) according to the methodology protected by the patent family WO2015185782A1 and described by Torras et al. in 2016 [11]. Briefly, a 1 µm thick thermal SiO_2_ layer was grown by wet oxidation on both sides of 100 mm Ø, 300 µm thick P-type silicon wafers. Positive photoresist was spun onto the wafers and exposed to UV light through a photomask to define the shape and lateral dimensions of the chips and baked. Then, dry-etching with C2F6 and CHF3 mixture [11] was applied to engrave the chips and the photoresist was stripped by plasma etching. Finally, feet underneath each chip were defined by Reactive Ion Etching with SF6 and C4F8 atmosphere (Figure 11).

For SPAchips^®^ functionalization with fluorophores, the methodology described previously [11] was followed with minor modifications protected by industrial secrets. Amine-terminated self-assembled monolayers (SAMs) were prepared as linkers to bind fluorophores onto the chips. To that aim, wafer surface was activated by plasma and then immersed in an ethanolic solution of (3-aminopropyl)triethoxysilane (APTES) (Merck, Darmstadt, Germany). After curing (110 °C, 1 h), silanized wafers were rinsed in ethanol and deionized water prior to fluorophore attachment by microcontact printing (µCP). To this aim, fluorophore printing solutions with Alexa Fluor^TM^ 488 NHS Ester and CF^TM^ 568 NHS (ThermoFisher Scientific, Waltham, MA, USA) were prepared in bicarbonate buffer 10 mM pH 9.0. Poly di-methyl siloxane (PDMS, Sylgard 184, Dow Corning, Midland, Michigan, USA) stamps were prepared by casting uncured PDMS on culture dishes and curing it at 75 °C for 1 h. PDMS stamps were activated with oxygen plasma (Harrick Plasma, Ithaca, NY, 30 W 1 min) and inked with 10 µL/cm^2^ of printing solutions and then applied on silanized SPAchips^®^ wafers for 10 min at room temperature. Excess of fluorophores was rinsed with an incubation with ethanolamine (ThermoFisher Scientific) and deionized water. Quality control was performed by fluorescence microscopy (Leica DM4 B). Images from 3 random areas per cm^2^ of wafer were taken and the chip mean fluorescence was analyzed to ensure than less of 10% of coefficient of variation was achieved per batch (Figure S2A,B). Finally, SPAchips^®^ were released with a mounting medium as described previously [11] and collected in individual sterile 1.5 mL tubes. Prior to chip assay, SPAchips^®^ were resuspended in culture medium according to manufacturer’s instructions at different selected concentrations (approximately 20,000 chips/µL) and used to label the primary cultures of neural and glial cells.

### 4.2. Isolation and Primary Cultures of Rat Macroglial and Microglial Cells

**Figure 12:**
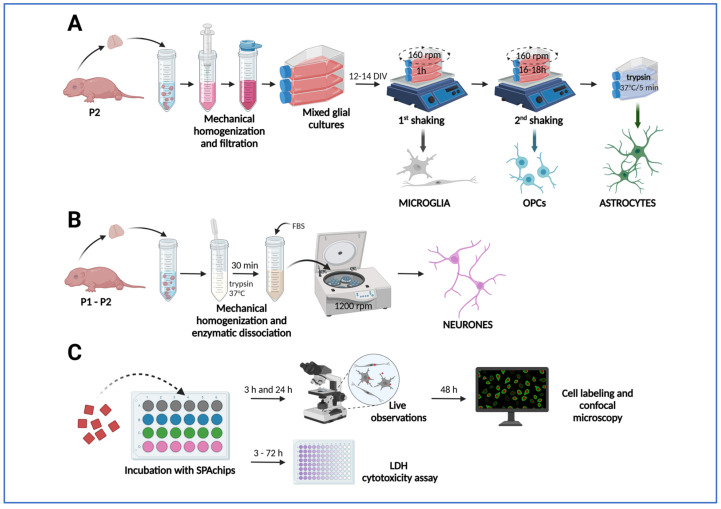
Application of SPAchips^®^ to primary cultures or rat glial and neuronal cells. (**A**) Establishing primary mixed glial cultures from brain hemispheres of neonatal rats and isolation of glial cell types by shaking-off method to obtain microglia and OPCs and mild trypsinization to obtain astrocytes. (**B**) Isolation of primary cortical neurons. (**C**) Culturing cells with addition of SPAchip^®^ and performing analyses to evaluate their effect. The arrows in the diagram show the successive stages of the experiments. Created in BioRender (latest version of web application). https://BioRender.com/huimaqj (accessed on 5 October 2025).

To obtain the particular fractions of macroglia (i.e., astrocytes and oligodendrocytes) and microglia, the brains of neonatal rats (n = 36) were isolated and used to establish primary rat mixed glial cultures according to the protocol described in detail elsewhere [73,74]. Briefly, mixed glial cultures were established from brain hemisphere of 1–2 days old Wistar rats, mechanically homogenized, and plated onto T75 culture flasks (Thermo Fisher Scientific) in culture medium composed from DMEM Glutamax (Gibco, Waltham, MA, USA), 10% of heat-inactivated fetal bovine serum (Gibco), and 1% antibiotic-antimycotic solution (Sigma, Burlington, MA, USA). After 12–14 days, flasks were shaken in an orbital shaker placed in an incubator for 1 h in order to detach microglia cells and then shaken overnight to detach oligodendrocyte progenitor cells (OPCs). Remaining astrocytes were subjected to mild trypsinization (5 min, 37 °C, Trypsin-EDTA (0.05%), Gibco; Figure 12A). Collected microglia, OPCs, and astrocytes were plated onto poly-L-lysine (PLL)-coated Flux coverslips (SPL) placed in 24-well plates (ThermoScientific). Microglia were plated at a density of 4 × 10^4^/cm^2^ and OPCs were plated at a density of 2 × 10^4^/cm^2^, both in culture medium composed of DMEM Glutamax (Gibco), 1% Insulin-Transferrin-Selenium supplement (Gibco), and 1% antibiotic-antimycotic solution (Sigma). Astrocytes were plated at a density of 4 × 10^4^/cm^2^ in culture medium composed of DMEM Glutamax (Gibco) and 1% antibiotic-antimycotic solution (Sigma). Glial monocultures were maintained in a humidified culture incubator at a temperature of 37 °C. To mimic the physiologically normoxic conditions, relevant to the characteristics of the nervous tissue, for culturing the particular cell fractions, the cell incubator equipped with the solid Zirconia O_2_ sensor was used for the precise control of physiological oxygen levels (the IncuSafe MCO-170 M Multigas Incubator, PHCBI, Chiyoda-ku, Tokio, Japan). The steady levels of O_2_ and CO_2_ were established at 5% and the potential oxygen excess was eliminated by flux of gas mixture (95% N_2_/5% of CO_2_).

### 4.3. Establishing Primary Cultures of Rat Neurons

Primary neuronal cultures were prepared from the cerebral cortex of newborn Wistar rats (post-natal day 1–2). Briefly, rats were decapitated and the cortex was isolated and triturated in Hanks’ Balanced Salt Solution (HBSS) (Gibco) by pipetting up and down. The cell suspension was incubated in 0.05% trypsin (Gibco) in HBSS at 37 °C to further dissociate the cells (Figure 12B). After 30 min, fetal bovine serum (FBS) (Invitrogen, Waltham, MA, USA) was added to a final concentration of 5% and the cell suspension was centrifuged at 1200 rpm. Pellet was then re-suspended in Neurobasal medium (Gibco) supplemented with 5% heat-inactivated bovine calf serum (Gibco), MACS^®^ NeuroBrew^®^-21 supplement (Miltenyi Biotec, Bergisch Gladbach, Germany) supplement, and 1 mM L-Gln, penicillin (100 u/mL), and streptomycin (100 μg /mL) (ThermoScientific). Neurons were plated at a density of 10^5^ cells/mL on the coverslips coated with poly-D-lysine (PDL; ThermoScienific). Then 2.5 μM of cytosine arabinoside (AraC) (Sigma-Aldrich) was added to the cultures on the second day after plating to inhibit the proliferation of non-neuronal cells. After 5 days, cells were incubated to the endpoint of the experiment in Neurobasal medium supplemented with 1 mM L-Gln, B27 supplement (ThermoScienific, Waltham, MA, USA), penicillin (100 μg /mL), and streptomycin (100 μg /mL).

### 4.4. Incubation with SPAchip^®^

SPAchips^®^ (A4cell Nanodevices, Cambridge, MA, USA) were added to OPCs 3 h after plating and to microglia and astrocytes 24 h after plating. Neurons were cultured up to 10–12 DIV before incubation with SPAchip^®^. SPAchips^®^ suspended in a sample buffer were counted in Bürker chamber (Supplementary Figure S1A) and added directly to the cultures in different volumes to obtain different concentrations per well (3 × 10^3^, 30 × 10^3^, or 150 × 10^3^ SPAchips^®^). Cultures were visually examined under an inverted microscope under transmitted light and fluorescence in CellObserver system (Zeiss) after 3 h and 24 h of incubation with SPAchips^®^ (Figure 12C).

### 4.5. Culturing the Retina Explants

Retinal explants were prepared as described previously [75]. C57Bl/6J (The Jackson Laboratory) were kindly provided by Dr. Catalina Hernández (CIBMS-CSIC, Madrid, Spain). After enucleation, neuroretinas were dissected from the eyes free of other tissues. Then, they were whole-mounted with the photoreceptor side up or down onto PTFE inserts (PICM03050, Millipore, Burlington, MA, USA). A total of 3 µL of chemically defined medium R16 [76] containing SPAchips^®^ (20,000 chips/µL) was added on each retina and then retinas were maintained in R16 [76] for 72 h at 37 °C in a 5% CO_2_ atmosphere. After the incubation time, retinas were fixed with 4% paraformaldehyde in 0.1 M Sorenson’s buffer (pH 7.4) for 1 h at room temperature and rinsed and stored.

### 4.6. LDH Assay

In order to evaluate the impact of incubation of different concentrations of SPAchips^®^ with cells on their viability, lactate dehydrogenase (LDH) release assay was performed. Lactate dehydrogenase is released into the culture medium with changes in cell membrane integrity and cell damage. LDH was evaluated with CyQUANT^TM^ LDH Cytotoxicity Assay (Invitrogen). The amount of 50 µL of culture medium was collected from culture wells on selected time points, 3 h, 24 h, 48 h, and 72 h, and frozen at −20 °C. The reaction was performed in a 96-well plate according to the protocol provided by the manufacturer. Absorbance was measured at 490 nm and 680 nm using a Fluostar Omega (1.30 software version) spectrophotometer (BMG Labtech, Ortenberg, Germany). Changes in LDH levels over time were estimated based on read absorbance values and normalized to the absorbance values measured at the first time point (3 h after starting incubation with SPAchips^®^) for each concentration of SPAchips^®^ in all the analyzed types of primary cultures.

### 4.7. Immunofluorescence Labelling

SPAchips^®^, counted with the use of Burker chamber (Supplementary Figure S1A), were added to the primary cultures of rat neonatal neural cells. Cell cultures were fixed after 48 h of incubation with OPCs and microglia and astrocytes were washed for 5 min in PBS (phosphate-buffered salt solution, Gibco), fixed for 15 min in 4% PFA (paraformaldehyde, Sigma), than washed twice for 5 min in PBS. Neuronal cultures were washed twice with TBS and fixed at 22–25 °C for 10 min with 100% cold methanol. For quick examination of the presence of SPAchips^®^ in the fixed cell cultures, cells were labelled with Hoechst 33342 solution (Sigma, 1:5000, 15 min) to visualize cell nuclei and observed in Zeiss LSM780 confocal microscope (Supplementary Figure S1B–E). For identification of cell phenotype, after fixing, OPCs, microglia, and astrocytes were blocked with 10% NGS (normal goat serum, Gibco) in PBS with 0,1% Triton (Serva, Heidelberg, Germany) for 1 h at room temperature. Neurons were permeabilized with 0.1% Triton X-100 in PBS for 15 min and then incubated for 1 h in blocking buffer (5% goat serum). Primary antibodies in 5% NGS were incubated overnight in 4 °C. Microglia cultures were labelled with classical markers of microglia/macrophages, including: OX42 (abcam, Cambridge, UK, ab1211; 1:200), ED1 (BioRad, Hercules, CA, USA, MCA341R; 1:400) and Iba1 (abcam ab107159; 1:400). OPC cultures were double labelled for oligodendrocyte lineage markers: MBP (Merck MAB381; 1:100) and OLIG2 (Merck, Darmstadt, Germany, AB15620; 1:500). Additionally, oligodendroglia were labelled for selected microglia and astrocyte markers, as those cells are often found in low percentages in OPC cultures. Likewise, OX42 (abcam ab1211; 1:200) and GFAP (Dako, Santa Clara, CA, USA, Z0334; 1:200) antibodies were applied to verify culture homogeneity. Astrocyte cultures were labelled with GFAP (Dako, Z0334; 1:200) and GLAST (abcam ab416; 1:200), as well as with anti-MBP and anti-OX42, as mentioned earlier, to verify culture homogeneity. Cells of neuronal lineage were stained with anti-MAP-2 antibody (Invitrogen, Waltham, MA, USA; 1:500). After incubation with primary antibodies, cultures were washed three times for 5 min in PBS. The corresponding secondary antibodies conjugated with either AlexaFluor 488 (green) or 633 (white), diluted 1:500 in PBS, were added for 2 h at room temperature. After washing with the excessive amounts of PBS, cultures were incubated with the Hoechst 33342 solution as described earlier, washed again and then mounted on the microscope slides with fluorescence mounting medium (Dako). The fluorescence images were collected on an LSM 780 /ELYRA PS.1 (Axio Observer, Reuil-Malmaison, France) confocal microscope (Zeiss, Oberkochen, Baden-Württemberg, Germany) under a 40× magnification objective for representative images of all glial fractions or under a 20× magnification objective for collecting microglia images for cell counting. ED1+ cells, Iba1+ cells, and OX42+ cells were counted from 10 random fields of view. 

For retinal explants, retinas were first permeabilized in 2% Triton X-100 (Sigma-Aldrich, Burlington, MA, USA) in PBS 1X pH 7.4, then incubated in blocking buffer (Tris buffer saline, TBS, 0.1 M Tris-HCl buffer pH 7.6 containing 1M Gly, 1% Tween-20, and 5% Normal Goat Serum) for 1 h, and then labelled with antibodies against GFAP (abcam ab7260; 1:500) or Recoverin (abcam ab31928; 1:50) in blocking buffer, overnight at 4 °C. After washing the retinas in TBS with 0.5% Tween-20 (TBS-T), retinas were incubated with secondary antibodies (1:100) and Alexa^®^647-conjugated peanut-aglutinin (PNA) in blocking buffer containing 1 µg/mL DAPI for 1 h at room temperature. Finally, excess antibody was rinsed with TBS-T and retinas were observed by confocal microscopy (Leica TCS SP5, Wetzlar, Germany).

### 4.8. Statistical Analysis

Statistical analyses of the quantitative results were performed using GraphPad 9.0 software. LDH assay results were tested with Shapiro–Wilk test and analyzed with two-way ANOVA. The values shown in the graphs represent the mean ± standard deviation of 3 biological replicates. Statistically significant differences in the graphs are shown with asterisks: * *p* < 0.05; ** *p* < 0.01.

## Data Availability

All data is contained within the article and Supplementary Material.

## Supplementary Materials

The following supporting information can be downloaded at: https://www.mdpi.com/article/10.3390/ijms26199773/s1.

## Abbreviations

The following abbreviations are used in this manuscript:

ACSA-1/GLAST	astrocyte cell surface antigen-1
BBB	blood–brain barrier
CNS	central nervous system
GCL	ganglion cell layer
GDNF	glial-derived neurotrophic factor
GFAP	glial fibrillary acidic protein
iNOS	inducible nitric oxide synthase
IPL	inner plexiform layer
MAP2	Microtubule-Associated Protein 2
MBP	myelin basic protein
OLIG2	transcriptional factor characteristic for oligodendroglial lineage
OS/IS	outer/inner segment
PDL	poly-D-lysine
ROS	reactive oxygen species
RGC	retinal ganglion cell layer
SPAchips^®^	suspended planar array chips
VACNF	vertically aligned carbon nanofiber
