# Supplementary material for: SPAchips: Microparticles Used for the Selective In Vitro Labelling of Microglia
Source: Int J Mol Sci. 2025 Oct 8;26(19):9773. doi: 10.3390/ijms26199773 (PMC12525483; doi:10.3390/ijms26199773)
Supplement: Supplementary file 1 [file ijms-26-09773-s001.zip › ijms-3847720-supplementary.pdf]

Supplementary Information:

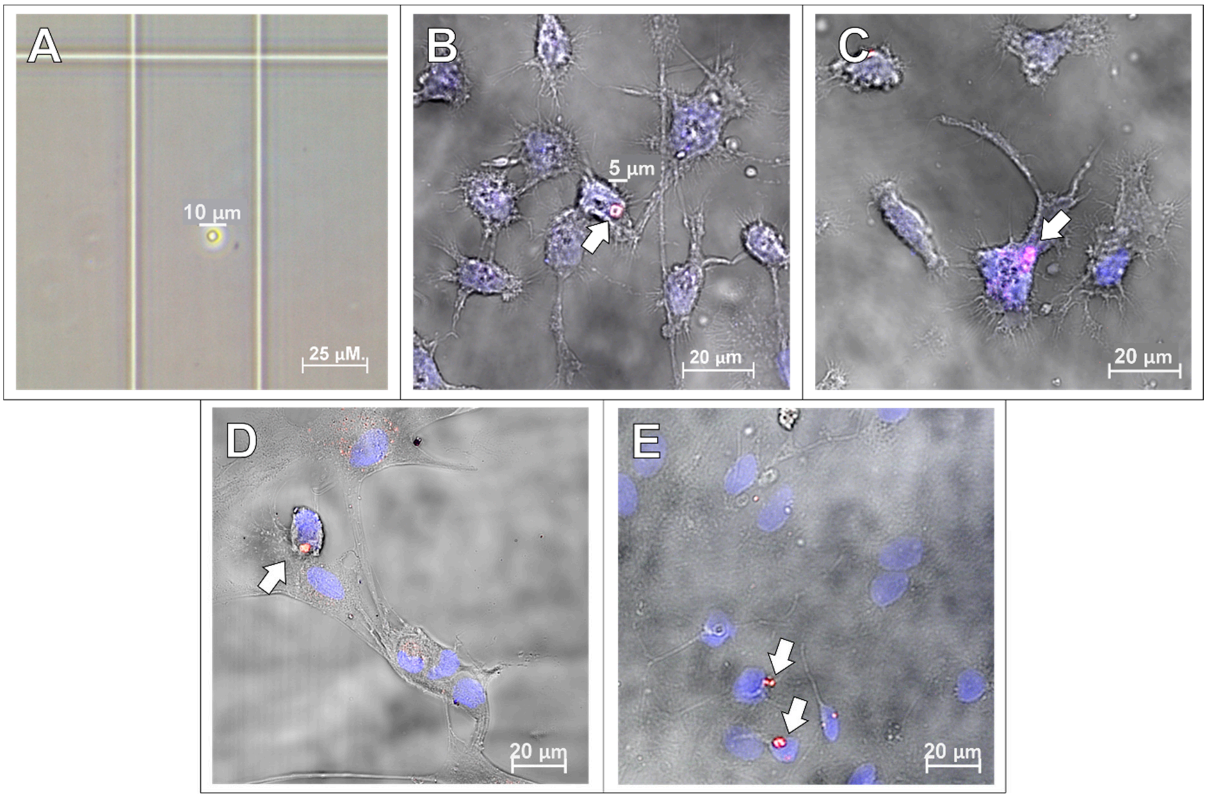

Figure S1: SPAchips® counting with the use of Burker chamber and AxioObserver under magnification 40x **(a)**. Cells fixed after 48 h of incubation with SPAchips® (3×10^3 chips per 8×10^4 cells), labelled with Hoechst cell nuclei marker and observed in Zeiss LSM780 confocal microscope under magnification 40x. White arrows indicate SPAchips® found in microglial cells **(b**,**c)**, single chip found in astrocyte culture **(d)**, and chips attached to cultured neurons **(e)**. The scale length in the image corresponds to 25 µm **(a)** or 20 µm **(b–e)**.

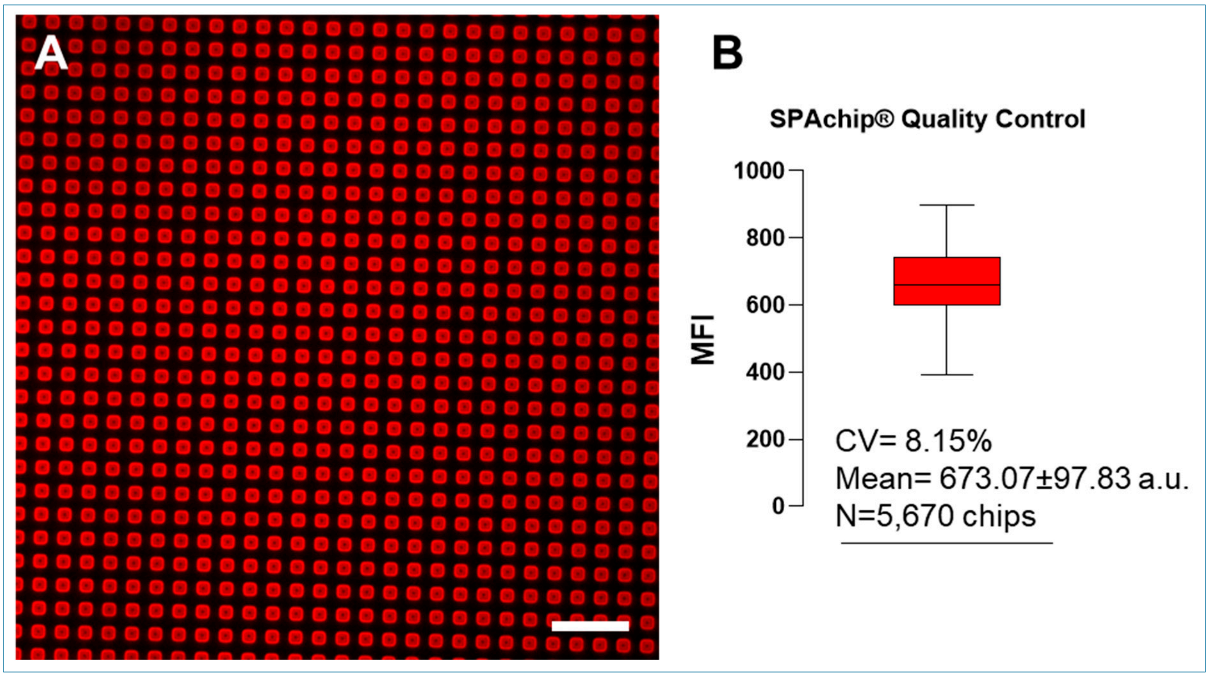

Figure S2: Example of a Quality Control assay performed with the SPAchip® used in this paper. **(A)** Representative image of a 50X field from a wafer functionalized with CF™568 fluorophore. Images of three random fields per cm2 of wafer were taken and Mean Fluorescence Intensity (MFI) of each chip was analysed. **(B)** MFI distribution (mean, quartiles and max to min values) of 5,670 chips analysed. To ensure homogeneous results in cells, coefficient of variation of SPAchip® fluorescence in each batch was less than 10%.
